# A versatile synthesis method of dendrites-free segmented nanowires with a precise size control

**DOI:** 10.1186/1556-276X-7-168

**Published:** 2012-03-05

**Authors:** Célia T Sousa, Diana C Leitao, João Ventura, Pedro B Tavares, João P Araújo

**Affiliations:** 1 IFIMUP and IN - Institute of Nanoscience and Nanotechnology, Faculty of Sciences, Universidade do Porto, Rua do Campo Alegre, 678, 4169-007, Porto, Portugal; 2 INESC-MN and IN - Institute of Nanoscience and Nanotechnology, Rua Alves Redol 9, Lisboa 1000-029, Portugal; 3 Dep. Química e CQ-VR, Univ. Trás-os-Montes e Alto Douro, Vila Real 5001-801, Portugal

## Abstract

We report an innovative strategy to obtain cylindrical nanowires combining well established and low-cost bottom-up methods such as template-assisted nanowires synthesis and electrodeposition process. This approach allows the growth of single-layer or multi-segmented nanowires with precise control over their length (from few nanometers to several micrometers). The employed techniques give rise to branched pores at the bottom of the templates and consequently dendrites at the end of the nanowires. With our method, these undesired features are easily removed from the nanowires by a selective chemical etching. This is crucial for magnetic characterizations where such non-homogeneous branches may introduce undesired features into the final magnetic response. The obtained structures show extremely narrow distributions in diameter and length, improved robustness and high-yield, making this versatile approach strongly compatible with large scale production at an industrial level. Finally, we show the possibility to tune accurately the size of the nanostructures and consequently provide an easy control over the magnetic properties of these nanostructures.

## 1 Introduction

**Figure 1:**
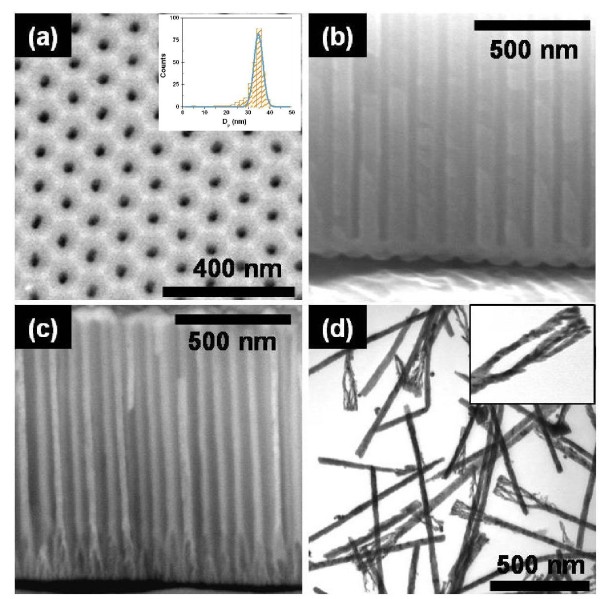
**SEM images of PAA template. (a) **PAA surface and **(b) **continuous oxide-layer present at the bottom of each nanopore in PAA. **(c) **SEM image of PAA template with Ni nanowires and **(d) **TEM images of suspended Ni nanowires with dendrites. TEM was performed in a LEO 906E Leica. Sample preparation included PAA dissolution in 0.4 M H_3_PO_4 _and magnetic separation, followed by a wash in bi-deionized water and finally re-suspended in 0.5 mL of ethanol.

Metallic and magnetic nanowires are a subject of relevant interest due to their promising applications in a diversified set of fields. The high aspect ratio of nanowires confer them outstanding properties and enhanced versatility promoting applications as nanosensors, nanoactuators, and nanocarriers [1,2]. In particular, multisegmented nanowires lead to new perspectives for surface functionalization, due to multiple available surfaces to target distinct particles/chemicals [3]. Various methods can be used for the preparation of single-layer and multi-segmented nanowires comprising different aspect ratios allowing one to fine tune their physical properties depending on particular requirements [4-6]. One elegant physicochemical route to prepare multifunctional nanostructures involves electrodeposition within the cylindrical nanopores of the self-assembled porous anodic alumina (PAA) [7,8]. However, the thick insulating alumina layer present at the bottom of the pores prevents uniform electrodeposition. Notice that this barrier layer is intrinsic to the anodization process and therefore needs to be removed or reduced before metallic nanowires can be grown (Figure 1b). To obtain high density arrays of nanowires using direct current (DC) electrodeposition method, the PAA is commonly detached from the Al substrate and the oxide barrier layer removed from the PAA using chemical or physical etching [9-11]. Afterwards, a metallic contact has to be deposited on one side of the free-standing PAA, which will then act as the working electrode [12]. Still, and due to the required laborious template processing, this method has its applicability restricted to free-standing thick membranes (> 20 *μ*m) [13], being therefore less suitable for industrial applications. Nevertheless, large scale production is highly desirable and for particular applications such as nanowires internalization in cells [14] or sensors comprising segmented nanowires [15], structures with shorter lengths (< 1 *μ*m) are crucial. A solution relies on the pulsed electrodeposition (PED) method that offers the possibility of growing nanowires with lengths ranging from few nanometers to several micrometers in PAA [8]. To perform PED in PAA, the insulator alumina layer has to be thinned, and the Al substrate will works as cathode [16,17]. The thinning process consists of a non-steady-state anodization using an exponentially decreasing anodization potential. As a result a reproducible tree-like branched structure is formed [18]. The main advantages of PED rely on its flexibility to adjust the amplitude and duration of the voltage pulses, offering the possibility to introduce a delay pulse to refresh the metallic ions concentration at the deposition interface, promoting uniform nanowire growth [8]. The unique disadvantage of PED in PAA templates is therefore the unavoidable presence of dendrites at the template bottom [19]. The latter when filled, originate ramified structures at one-end of the nanowires, altering their physical properties, and hinder the biomedical applications since the small dendritic structures can be released and became toxic [12].

In this letter, we report an innovative strategy to fabricate dendrite-free nanowires ranging from few nanometers to several micrometers in length. The introduced approach suppresses the main disadvantage of homogeneous pore filling of PAA using PED, i.e., the dendritic structures are selectively etched, leading to nanowires free from end-ramifications. Here, single-layer and segmented nanowires with well controlled dimensions, showing a narrow size distribution (both in diameter and length) were successfully prepared. We support the robustness of the presented method providing the influence of the nanowires aspect ratio on their magnetic properties.

## 2 Experimental

PAA templates were fabricated using a two-step anodization process [20], in 0.3 M (COOH)_2 _at 4°C, under 40 V. The nanopores display 35 nm in diameter, 105 nm of interpore distance, and length of 1.5 *μ*m (pore growth rate of 2.5 *μ*mh^-1^) as shown in Figures 1a, b. After the second anodization, the alumina barrier layer at the bottom of the pores shows a thickness of ~ 52 nm, preventing the PED process. Thus, this barrier layer was smoothly thinned by applying an exponentially decreasing voltage from 40 V down to 8 V. This non-steady-state anodization originates tree-like branched pores with various lengths and diameters [19]. The final potential value corresponds to a barrier layer thickness of 10 nm as obtained from [21], providing the conditions for which the most uniform nanowires length is attained [22].

Subsequently, the PAA is filled with the desired material by PED method [22,23]. In this study, we use Ni, Au and Cu: Ni was deposited at 40°C from an aqueous solution containing NiSO_4_.6H_2_O (300 g/L), NiCl_2_.6H_2_O (300 g/L) and H_3_BO_3 _(45 g/L); Au nanosegments were deposited from a 2.5 DWT/Qt. Technic, Inc. electrodeposition solution at 40°C; and Cu was deposited at room temperature from CuSO_4_.5H_2_O (1 M) H_3_BO_3 _(45 g/L). Segmented nanowires were prepared by first depositing Cu in the branched pores, followed by Au and finally Ni. The three materials were grown using a cycle with three different pulses. Initially, the material is deposited by applying a constant current pulse (70 mA/cm^2^) for 8 ms. Then, a second pulse with opposite polarity and constant potential of 8 V is used during 2 ms. This pulse is used to discharge the barrier-layer (which acts as a capacitor). It also homogenizes the membrane, helping to repair small cracks that may appear during the first step. Finally, a rest pulse of 0.7 s is applied, introducing a delay time to refresh the metallic ions concentration at the deposition interface and promoting a uniform growth of nanowires. Notice that the dendrites were filled in 55 s, whereas the length of the Au and Ni segments can be varied using the calculated deposition rates of 2.0 nm/s and 1.5 nm/s, respectively. In addition, and when required, the Al substrate was removed with 0.5 M CuCl_2 _and 10% HCl. The PAA template was dissolved in an aqueous solution of 0.4 M H_3_PO_4 _and 0.2 M H_2_Cr_2_O_7_. To selectively etch the Cu dendrites a 1% HNO_3 _solution was used.

## 3 Results and discussion

Figures 1a, b show top and cross sectional SEM images of PAA, respectively. Inherent to the process of PAA growth is the presence of a thick continuous oxide-layer at the bottom of each nanopore (Figure 1b) [22,24]. Therefore, after the second anodization the barrier layer was thinned and the metal electrodeposited. Figure 1c shows single-layer Ni nanowires embedded in a 1 *μ*m thick PAA template. As seen, the employed barrier layer thinning procedure leads to ~300 nm long non-uniform branched pores at the bottom of the template, which is subsequently replicated by the Ni nanowires. The presence of such structures with several diameters and lengths leads to the loss of the major advantage of this template assisted method, i.e., the nanowires homogeneity in size. Even, after removing the PAA template by chemical etching, the free-standing nanowires remain with the dendrites at the bottom end as shown in the TEM image (Figure 1d). These inhomogeneities confer distinct physical properties and may lead to anomalous behaviors when compared to the main cylindrical nanowires [25]. Such drawback strongly limits practical applications combining PAA and PED to attain extremelly small features.

**Figure 2:**
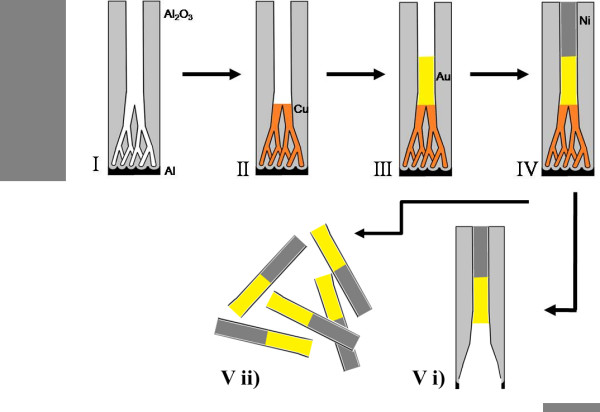
**Scheme of the strategy PAA selective filling and dendrites removal**.

**Figure 3:**
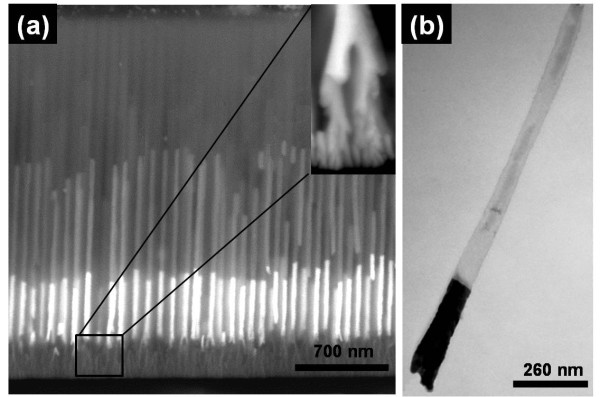
**SEM and STEM images of nanowires. (a) **SEM image of PAA filled with Cu in dendrites (branched pores at the pore bottom) and Au-Ni segmented nanowires, with 400 and 800 nm, respectively and **(b) **STEM image of a suspension of dendrites free Au/Ni segmented nanowires.

A straightforward strategy was successfully developed to overcome the intrinsic disadvantages of using PED to fill PAA templates. Figure 2 illustrates the basic steps behind this approach. After fabricating the PAA and thinning the barrier layer [step I], the non-homogeneous branched pores at the bottom are filled with Cu, an easily soluble material [step II]. The PED conditions were optimized to increase the nucleation rates, leading to a homogeneous length in the dendrites filling [26]. Achieving such high degree of uniformity is crucial as it is later reflected on the control of the main cylindrical nanowires thickness [22]. Then, the main PAA pores are filled with any other material, as long as it can survive the selective etching of the dendrites [step III]. Here we choose Au and Ni, thus combining the magnetic properties of Ni with the different surface coordination chemistry of Au/Ni towards a selective functionalization [10]. However, others materials like Ag or Pt can also be used as a metallic segment and Co, NiFe or Fe as a magnetic segment. The most striking feature of this method is that either a single-layer or several layers of distinct materials can be deposited inside the PAA pores, creating customized segmented nanowires [step IV]. Furthermore, and since for some industrial applications there is great interest in producing large quantities of nanowires, our strategy provides an ingenious route to fabricate several generations of nanowires through the electrodeposition of a sacrificial material between the metallic layers of interest allowing the fabrication of several series of nanowires in the same PAA template. Finally, the undesirable features (dendrites and intermediate layers; e.g., Cu layer) can be easily removed by selective dissolution, in an elegant and expedite way. This step can be performed with the nanowires either inside the PAA template [step V(i)], where the Al is only partially removed in the area just adjacent to the nanowires bottom, enabling dendrites dissolution. On the other hand, free nanowires can also be obtained [step V(ii)] by removing completely the PAA template, being the dendrites subsequently dissolved in the nanowires suspension. Interestingly, in the last case [step V(ii)] the Al substrate is preserved and can be used to repeat the described procedure several times (first anodization; second anodization and thinning; electrodeposition; dissolution). Notice that, so far, perfect cylindrical nanowires were restricted to several *μ*m long after intricate processing steps, thus preventing the broader use of the nanowires at an industrial scale. On the contrary, our strategy allows the growth of cylindrical nanowires with lengths down to few nanometers due to the crucial combination of PAA and PED methods that in turn enable a precise control over the length and diameter of the nanowires. Figure 3a shows Au/Ni bilayer nanowires with Cu dendrites embedded in the PAA template. The optimized growth conditions used for the multilayered nanowires result in an extremely uniform length of the different segments (Figure 3a). The length of the segments can be customized by changing the electrodeposition time; in contrast, its homogeneity is mainly dictated by the uniformity of the thin oxide layer at the bottom of the nanopores [22]. The cross sectional SEM image in Figure 3a shows the dendrites completely filled with Cu, then a Au layer with a thickness of ~400 nm, and finally a Ni layer on top with ~800 nm thickness. Histogram of the nanowires diameter distribution was obtained from the statistical analysis of SEM images. The inset of Figure 1a shows representative nanowires size distribution in length. Recall that the nanowires replicate the PAA pores, and thus the size distribution in diameter is identical to that obtained for the PAA pore. Also the analysis performed on cross-section SEM images of the nanowires (Figure 3a) still embedded in the PAA confirms the homogeneity along the nanowires. The obtained Gaussian distributions display well defined peaks with average value (standard deviations) of 34.4 nm (2.1 nm) in nanowire diameter. These results correspond to a low dispersion of the nanowires sizes and translate an improved uniformity when compared with reported data in the literature [27,28]. After the PAA removal, the segmented nanowires were resuspended and the Cu etched away (Figure 3b). The Cu dendrites were successfully removed, leaving clean ended nanowires. Clearly, and although the Au/Ni interface is expected to be a fragile spot of these nanowires, the structures are seen to withstand the process of dissolution/washing showing mechanical robustness, and thus making this new approach extremely viable for high-yield synthesis.

**Figure 4:**
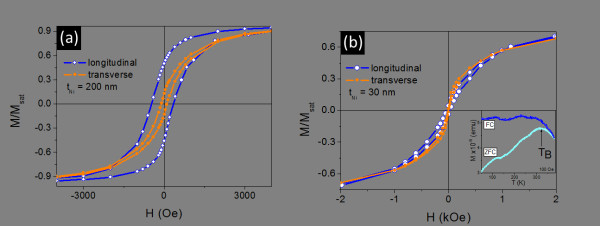
**M(H) curves at 300 K with magnetic field applied parallel and trans-verse to the nanowires longitudinal axis for Ni nanowires of (a) 200 nm and (b) 30 nm**. Inset shows ZFC and FC magnetization measurements of Ni nanowires with 30 nm length.

Figure 4 shows the magnetization versus applied field [M(H)] measurements for PAA embedded double segmented nanowires [Au (200 nm)/Ni(30 or 200 nm)] with Cu filled dendrites. These curves were taken at 300 K with the magnetic field applied parallel and transverse to the nanowires longitudinal axis. For the nanowires with thicker Ni elements (200 nm) (Figure 4a), we observe an anisotropic M(H) behavior [25], consequence of the high-aspect-ratio (length/diameter ~6) that leads to a strong shape anisotropy with well defined magnetic easy-axis lying along the nanowires longitudinal direction. The coercive fields along the longitudinal and transverse directions were found to be 400 and 100 Oe, respectively. On the other hand, for the thinner Ni layer (30 nm), similar M(H) cycles are observed in both directions (Figure 4b). Temperature dependent magnetization M(T) measurements in the Ni nanowires with 30 nm length under zero-field-cooling (ZFC) and field-cooling (FC) are also presented (inset Figure 4b). The ZFC magnetization curve shows a maximum, with a bifurcation between FC and ZFC data below a certain temperature. This behavior is typically present in an assembly of ferromagnetic nanoparticles exhibiting superparamagnetism above a certain temperature [29], as expected for Ni nanoparticles with diameter below 50 nm, for which superparamagnetism has been reported previously [30]. The peak observed in the ZFC curve is associated with the blocking temperature (T_B_) of ~315 K (inset Figure 4b). Therefore, since the M(H) curves presented in Figure 4b were obtained at 300 K, a low coercive field is observed (< 30 Oe). Such easiness in adjusting the nanowires geometrical dimensions, allow us the effective possibility to tune the magnetic properties of the fabricated nanowires green from a ferromagnetic to to a superparamagnetic green regime.

## 4 Conclusions

In summary, we demonstrated an innovative and expedite method to grow cylindrical nanowires in a controlled way ranging from nanometers to micrometers in length. With this method the major limitation of using PAA templates is overcome by selectively removing the dendritic ends, formed due to the inherent barrier layer thinning step required for nanowires growth. Therefore, the presence of undesirable contributions to the magnetic response which may arise from such non-homogenous features are easily removed. Also, several materials can be used to synthesize these nanostructures as long as the dendrites can be chemically etched without affecting the main nanowires dimensions and composition, evidencing the enormous versatility of the presented strategy. We successfully combined well established top-down methods such as PAA template-assisted nanowire synthesis and electrodeposition, which have displayed a large appeal for industrial processing due to their lower-cost and higher-yield. Finally, considering the growing industrial interest in magnetic nanoparticles and high-aspect-ratio segmented nanowires, the developed approach enables the synthesis of nanostructures with high homogeneity in diameter and highly controllable length allowing an effective tune of their magnetic behaviors ultimately enabling reaching the superparamagnetic regime.

## Competing interests

The authors declare that they have no competing interests.

## Authors' contributions

CTS carried out the samples preparation, the SQUID measurements the SEM analysis, and drafted the manuscript; DCL participated in drafted of the manuscript and performed the statistical analysis of the SEM and TEM images; JV performed the magnetic measurements analysis and participated in the sequence alignment; PBT carried out the TEM measurements and JPA participated in the magnetic measurements analysis and conceived of the study, and participated in its design and coordination. All authors read and approved the final manuscript.
